# A tonoplast Glu/Asp/GABA exchanger that affects tomato fruit amino acid composition

**DOI:** 10.1111/tpj.12766

**Published:** 2015-02-24

**Authors:** Christopher J. Snowden, Benjamin Thomas, Charles J. Baxter, J. Andrew C. Smith, Lee J. Sweetlove

**Affiliations:** ^1^ Department of Plant SciencesUniversity of OxfordSouth Parks RoadOxfordOX1 3RBUK; ^2^ Central Proteomics FacilitySir William Dunn Pathology SchoolUniversity of OxfordSouth Parks RoadOxfordOX1 3REUK; ^3^ SyngentaJealott's Hill International Research CentreBracknell, BerkshireRG42 6EYUK

**Keywords:** amino acid, ripening, tomato, transport, vacuole

## Abstract

Vacuolar accumulation of acidic metabolites is an important aspect of tomato fruit flavour and nutritional quality. The amino acids Asp and Glu accumulate to high concentrations during ripening, while γ‐aminobutyrate (GABA) shows an approximately stoichiometric decline. Given that GABA can be catabolised to form Glu and subsequently Asp, and the requirement for the fruit to maintain osmotic homeostasis during ripening, we hypothesised the existence of a tonoplast transporter that exports GABA from the vacuole in exchange for import of either Asp or Glu. We show here that the tomato vacuolar membrane possesses such a transport property: transport of Glu across isolated tonoplast vesicle membranes was *trans*‐stimulated in counterexchange mode by GABA, Glu and Asp. We identified SlCAT9 as a candidate protein for this exchanger using quantitative proteomics of a tonoplast‐enriched membrane fraction. Transient expression of a SlCAT9‐YFP fusion in tobacco confirmed a tonoplast localisation. The function of the protein was examined by overexpression of SlCAT9 in transgenic tomato plants. Tonoplast vesicles isolated from transgenic plants showed higher rates of Glu and GABA transport than wild‐type (WT) only when assayed in counterexchange mode with Glu, Asp, or GABA. Moreover, there were substantial increases in the content of all three cognate amino acids in ripe fruit from the transgenic plants. We conclude that SlCAT9 is a tonoplast Glu/Asp/GABA exchanger that strongly influences the accumulation of these amino acids during fruit development.

## Significance Statement

Tomato fruit undergo major shifts in amino acid composition during ripening with the quantitatively most important changes being a decline in GABA and a stoichiometric increase in Asp and Glu. We have identified a tonoplast transporter, SlCAT9, that facilitates the counter‐exchange of GABA / Glu / Asp across the fruit vacuolar membrane and strongly influences the levels of the three amino acids in ripe fruit.

## Introduction

Developmental changes in metabolite composition are of crucial importance for the function of fruits as seed dispersal systems; metabolite composition determines the palatability of the fruit, encouraging consumption of the fruit only when it is fully ripe. For crops such as tomato, metabolite composition is also a key facet of fruit quality both with respect to flavour and nutritional quality. In addition to well characterised changes in sugar and carboxylic acid content, tomato fruits also undergo major changes in amino acid composition during development (Klee and Giovannoni, 2011). The most quantitatively important amino acids are GABA, Glu and Asp. GABA accumulates during the initial phase of fruit development, with GABA content peaking when the fruit has reached its maximum volume. As the fruit ripens, GABA content declines and Glu and Asp accumulate (Figure S1; Rolin *et al*., 2000; Carrari *et al*., 2006).

**Figure 1:**
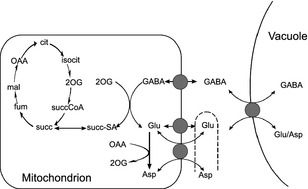
Metabolic interconversion of GABA and glutamate during fruit ripening. Pathway diagram showing the metabolic interconnections between GABA and Glu. Some reactions are omitted for clarity (e.g. mitochondrial glutamate dehydrogenase, pyruvate‐dependent GABA transaminase and cytosolic glutamate decarboxylase). The mitochondrial transporters shown are a GABA permease, a glutamate carrier and an aspartate‐glutamate exchangers. 2OG, 2‐oxoglutarate; cit, citrate; fum, fumarate; isocit, isocitrate; mal, malate; OAA, oxaloacetate; succ, succinate; succ‐SA, succinic semialdehyde.

Because the decline in GABA is approximately stoichiometric with the increase in Glu and Asp, it is likely that GABA is interconverted into the latter two amino acids and there is an established metabolic route by which this can occur in mitochondria (Koike *et al*., 2013) (Akihiro *et al*., 2008; Yin *et al*., 2010). The relatively high amounts of these amino acids that accumulate in tomato fruit [e.g. ripe tomato contain 10 mmol Glu kg^−1^ fresh weight (Sorrequieta *et al*., 2010)] means that within the highly vacuolated cells in the pericarp (Johnson *et al*., 1988), they must be stored mainly in the vacuole. Hence, the developmental exchange of GABA for Glu and Asp requires export of GABA from the vacuole into the cytosol, transfer into the mitochondrion for metabolism into Glu and Asp, followed by export of Glu and Asp from mitochondrion to cytosol and finally uptake into the vacuole (Figure 1).

Apart from a mitochondrial GABA permease that has been identified in Arabidopsis (Michaeli *et al*., 2011), the transporters required for these intracellular transport events are not known. Based on homology with other eukaryotes, plant mitochondria are predicted to possess both a Glu carrier (GC) and an Asp–Glu exchange carrier (AGC), although neither of these have yet been identified at the molecular level from amongst the plethora of candidate genes in the mitochondrial carrier family (Palmieri *et al*., 2011). Moreover, almost nothing is known about the transport of amino acids across the tonoplast in plants (Shiratake and Martinoia, 2007; Martinoia *et al*., 2012; Tegeder, 2014). Proteomic studies of vacuoles isolated from *Arabidopsis thaliana* have so far revealed only a few candidate amino acid transporters (Carter *et al*., 2004; Endler *et al*., 2006; Jaquinod *et al*., 2007), and none has so far been investigated to determine which substrates they may transport. Within the cationic amino acid transporter (CAT) family, for example, several members have been shown to localise to the tonoplast in Arabidopsis (AtCAT2, AtCAT4, AtCAT8, AtCAT9), but the substrate selectivity and transport properties of these proteins remain largely untested (Su *et al*., 2004; Yang *et al*., 2010; Okumoto and Pilot, 2011).

The aim of this work, therefore, was to identify the tonoplast transporter(s) involved in the transport of GABA, Glu and Asp between vacuole and cytosol. The work also sought to test two hypotheses: first that a single exchange‐transporter could be responsible for the required movement of GABA and Glu/Asp. The argument for the presence of a single exchanger rather than separate tonoplast transporters for import and export of the different amino acids is that the ripening fruit must maintain vacuolar turgor while changing its vacuolar amino acid composition. A transporter that works in a strict counterexchange mode would ensure that the osmolarity of the vacuole and turgor balance is maintained. The second hypothesis we wished to test was that the exchanger would exert appreciable control over the abundance of Glu and Asp in ripe fruit and thus would represent a good engineering target for manipulating fruit flavour.

## Results

### Proteomic identification of the SL1.00sc04801_50.1.1 gene‐product as a candidate Glu/Asp/GABA transporter

We reasoned that tonoplast transporters involved in the accumulation of Glu and Asp during the latter stages of ripening might increase in abundance from mature green to breaker (when approximately 10% of the fruit is orange/red in colour) to red fruit. To analyse the composition of the tonoplast and identify putative Glu/Asp transporters based on abundance changes, we carried out a quantitative proteomic analysis of a tonoplast‐enriched membrane fraction (Figure S2) isolated from fruits at different stages of ripening. To provide a link between the proteomic analysis and data from a tomato introgression population (Eshed and Zamir, 1995; Overy *et al*., 2005; Schauer *et al*., 2006), fruit from the M82 cultivar were used. Purity of the membrane fraction was assessed by using inhibitors for ATPases of specific classes of ATPases. The majority of the mitochondrial ATPase (inhibited by azide) and plasma membrane ATPase (inhibited by vanadate) passed through the stepped sucrose gradient that we used and were found in the pellet (Figure S2a). ATPase activity in the tonoplast‐enriched membrane fraction (collected at the interface between the 6 and 12% sucrose steps) was mainly tonoplast ATPase (80% inhibited by nitrate) and contained only a small amount of mitochondrial ATPase (< 1% inhibited by azide) and no detectable plasma membrane ATPase (zero inhibition by vanadate). Moreover, the ATP‐dependent proton‐pumping activity of vesicles derived from this fraction was almost completely inhibited by concanamycin A, a specific inhibitor of the tonoplast ATPase (Figure S2c). Tonoplast‐enriched membranes were carbonate‐washed to remove peripheral proteins, and the quality of the protein fractions was assessed via SDS‐PAGE (Figure S2b). Proteins were identified in each fraction using shotgun proteomics and quantitative estimations of each identified protein obtained from spectral counts. We restricted our analysis to those proteins that appeared in both biological replicates and to proteins with at least two peptides identified. The data set was further restricted to integral membrane proteins using HMMTOP (Tusnady and Simon, 2001) for hydropathy prediction and was subsequently filtered to remove proteins homologous to those with published non‐tonoplast localisation. This screening left 96 proteins (Data S1). A further selection for those proteins exhibiting a consistent increase in abundance between mature green and red tonoplast in both replicates gave rise to a smaller data set. Table S1 lists the proteins from this set that are homologous to known transporters and shows their abundances within the two replicate sets. Two of the proteins are members of amino acid transporter families: SL1.00sc07184_335.1.1 (Solyc11g008440.1.1) and SL1.00sc04801_50.1.1 (Solyc10g018600.1.1).

Solyc11g008440.1.1 increases from an average of 0.02% of tonoplast protein at mature green stage to 0.06% in red fruit (Table S1) and is homologous to *Saccharomyces cerevisiae* Avt1p (NP_012534.1), a member of the Avt family of vacuolar transporters belonging to the amino acid/auxin permease (AAAP) family (TC 2.A.18) within the amino acid–polyamine–organocation (APC) superfamily of the Transporter Classification Database (Saier *et al*., 2009; Wong *et al*., 2012). The gene encoding SlAvt1 falls in the chromosomal area overlapped by introgression lines IL11‐1 and 11‐2. Based on the published introgression line metabolite data (Schauer *et al*., 2006), IL11‐1 is not a QTL for Asp, Glu, or GABA; however, IL11‐2 is a QTL for increased Asp and GABA, showing 1.70‐ and 1.62‐fold increases in the amounts of these two metabolites, respectively. As SlAvt1 is encoded by a gene in the overlapping region between the two lines, it is unlikely to be responsible for these traits.

The other candidate, Solyc10g018600.1.1, increases from an average of 0.02% of tonoplast protein at mature green stage to 0.12% in red fruit (Table S1) and is a homologue of *Arabidopsis thaliana* cationic amino acid transporter 9 (CAT9; At1g05940; NP_563754.1). Whilst members of the CAT subfamily of transporters predominantly transport cationic amino acids, plasma‐membrane‐localised members of this family from *Arabidopsis* have been shown to possess the capacity for the transport of both cationic and anionic amino acids (Su *et al*., 2004). Sequence analysis of the tomato and Arabidopsis CAT family members demonstrated that the CAT9 proteins from both species belong to a distinct monophyletic clade consisting of putative CAT9 homologues that is sister to a clade containing AtCAT2, AtCAT3 and AtCAT4 (Figure S3). The increase in abundance of SlCAT9 during ripening varied significantly between the two biological replicates, but in both cases its abundance was higher in red than green fruit. The gene encoding SlCAT9 falls within an introgression line with a QTL for increased GABA (Schauer *et al*., 2006). The combination of the GABA QTL with the increasing abundance of SlCAT9 during ripening made SlCAT9 a strong candidate for further study.

### SlCAT9 is a tonoplast‐localised protein that increases in abundance during fruit ripening

To confirm the tonoplast localisation of SlCAT9 and to investigate changes in protein abundance, a peptide antibody was raised against an immunogenic region (amino acids 24‐38: SSALRSKPLASPSET) of SlCAT9. To test the antibody, tonoplast extracts from mature green, breaker and red fruit were subjected to SDS‐PAGE and western blotting. Pre‐immune serum did not detect any proteins in the extracts, whilst the affinity‐purified antiserum recognised a single band at 50 kDa, consistent with the predicted molecular weight of SlCAT9 (Figure S4). This band increased in intensity across the developmental stages, matching the proteomic data.

**Figure 2:**
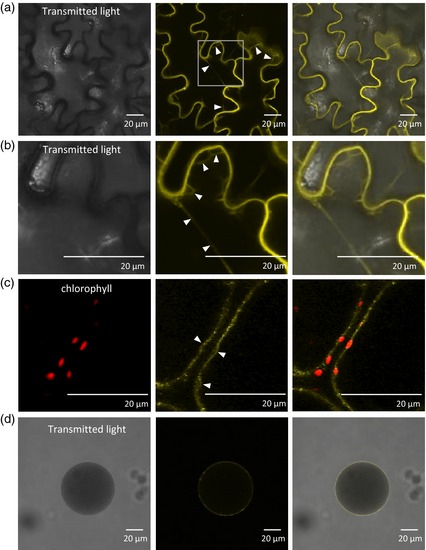
Intracellular localisation of SlCAT9. A chimeric fusion of SlCat9 and YFP was transiently expressed in *N. tabacum* leaf epidermal cells (a–c). Intact vacuoles were isolated from transiently transformed *N. tabacum* leaf tissue (d). Fluorescent proteins were visualised using confocal laser scanning microscopy. Arrowheads in (a, b) indicate *trans*‐vacuolar strands. (b) Is an enlargement of the region of (a) indicated by the white box in the middle panel of (a). (c) Shows tonoplast bulges (indicated by arrowheads) caused by chloroplasts visualised by chlorophyll autofluorescence.

To establish the localisation of *Sl*CAT9 a chimeric C‐terminal fusion of SlCat9 and YFP was generated and was transiently expressed in tobacco leaves. The YFP fluorescence pattern in tobacco epidermal cells as visualised by confocal laser scanning microscopy indicated a membrane localisation with the signal mainly restricted to a narrow zone close to the cell periphery (Figure 2). There were several fluorescent features that suggest the protein is localised to the tonoplast and not the plasma membrane. First, there were many transverse strands of intracellular fluorescence (Figure 2a,b) and these are most likely *trans*‐vacuolar strands. Second, there were several places were the fluorescence was displaced from the cell periphery as it bulges around organelles such as chloroplasts (Figure 2c). Furthermore, intact vacuoles isolated from transformed leaves contained the fusion protein in their tonoplast membrane, as was apparent from the fluorescence pattern (Figure 2d), confirming a tonoplastic localisation.

### Characterisation of transport properties of SlCAT9 by overexpression in transgenic tomato fruit

**Figure 3:**
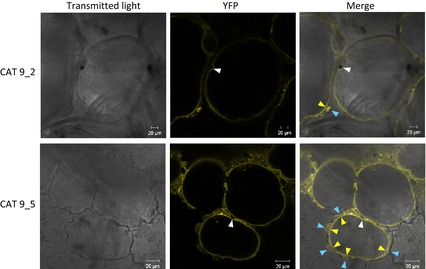
Localisation of the SlCAT9‐YFP fusion protein overexpressed in transgenic tomato fruit. YFP fluorescence was visualised in ripe‐red T_1_ transgenic fruit mesophyll cells using confocal laser scanning microscopy. Two independent transgenic lines, CAT 9_2 and CAT 9_5, are shown. White arrowheads indicate regions where the tonoplast bulges around an organelle. Yellow arrowheads indicate regions where the tonoplast is clearly separated from the cell periphery (blue arrowheads).

To examine the transport properties of SlCAT9 we initially attempted to reconstitute the recombinant protein into liposomes, but without success. We also tried making transport measurements in yeast expressing the protein, but were unable to obtain reproducible data. Given that Arabidopsis CAT proteins were also found not to be functional when expressed in *Xenopus* oocytes (Su *et al*., 2004), we decided instead to analyse the transport properties of SlCAT9 *in planta*, by making overexpressing transgenic tomato lines. Transgenic Micro‐Tom plants overexpressing a chimeric *SlCAT9‐YFP* gene were therefore generated. Overexpression was achieved using the ethylene‐inducible E8 promoter (Deikman and Fischer, 1988) to mimic the expression profile of the endogenous *SlCAT9*, reducing the risk of complications due to ectopic expression. Kanamycin‐resistant lines were screened for transgene expression in fruit at red stage using an anti‐GFP (green fluorescent protein) antibody to detect the YFP fusion protein. Two lines (CAT9_2 and CAT9_5) in which the fusion protein was present in the highest amounts (Figure S5a) were selected for further analysis. Confocal microscopy confirmed the presence of the YFP fusion protein in fruit mesophyll cells of both lines (Figure 3). Moreover, the fluorescence pattern provided further evidence for a tonoplast localisation of CAT9: there were clear instances where fluorescence was present in a membrane clearly separated from the cell periphery and additionally where the membrane was displaced inwards as it passed around a large organelle (probably the nucleus). A number of cytoplasmic punctate structures were also visible, most likely due to the presence of the protein in endomembrane compartments as it was trafficked to the tonoplast. Overexpression of SlCAT9‐YFP did not lead to any major growth phenotype in T_1_ plants, with fruit mass and seed number both unchanged (Figure S5b). However, there was a consistent difference in fruit morphology: fruit on the transgenic plants were heart‐shaped, rather than spherical as was the case with WT fruit (Figure S5c).

**Figure 4:**
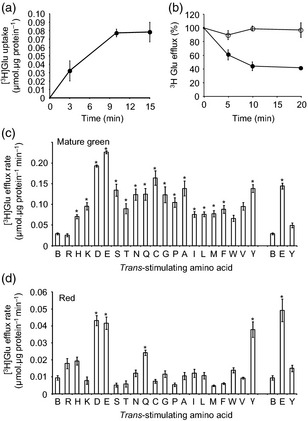
Glutamate transport characteristics of tomato fruit tonoplast vesicles. (a) Time‐dependent uptake of [^3^H]glutamate into tonoplast vesicles isolated from mature green fruit. Values are the means ± standard error (SE) of three replicates. (b) After 15 min of uptake vesicles were diluted in either buffer (unfilled symbols) or buffer containing 25 mm unlabelled glutamate (filled symbols) in order to stimulate [^3^H]glutamate efflux. The values are means ± SE of three replicates. The selectivity of glutamate transport was investigated using *trans*‐stimulation of glutamate efflux from mature green (c) and red (d) fruit tonoplast vesicles pre‐loaded with [^3^H]glutamate. Efflux rates in zero‐*trans* configuration and in *trans*‐stimulation configuration due to the presence of different unlabelled amino acids (25 mm) are shown. Tyrosine was added at a lower concentration (2.5 mm) to maintain solubility. Glutamate was also tested at this lower concentration for comparison. Amino acids are given as single letter codes with buffer alone (i.e. zero‐*trans* control) labelled as ‘B’ and GABA labelled as ‘γ’. Values are means ± SE from three biological replicates consisting each of three technical replicates. *Indicates significantly different from buffer control ‘B’; *t*‐test, *P* < 0.05.

The abundance profile of SlCAT9 suggested that it could be involved either in the efflux of GABA from the vacuole, the influx of Glu/Asp, or both together, operating as a Glu/Asp/GABA exchanger. To explore these possibilities, we isolated transport‐competent vesicles from fruit tissue and established a transport assay that allowed the measurement of exchange transport via *trans*‐stimulation, followed in this configuration as the enhancement of efflux of a radiolabelled substrate from pre‐loaded vesicles by addition of an external unlabelled substrate. This *trans*‐stimulation effect is the defining characteristic of transporters that operate in a counterexchange mode (Stein, 1990; Ma *et al*., 2012). It also provides a means of exploring the substrate selectivity of the transport system, allowing discrimination between solutes that are actually transported across the membrane and those that simply compete for access to the substrate‐binding site of the transporter. We initially established the assay using radiolabelled Glu. Tonoplast vesicles from WT fruit showed time‐dependent uptake of [^3^H]Glu (Figure 4a). Moreover, once net uptake was complete, addition of unlabelled Glu at saturating concentration to the external medium stimulated efflux of [^3^H]Glu from the vesicles (Figure 4b), demonstrating that a Glu exchanger is present in the tomato tonoplast. Note that although we are measuring [^3^H]Glu efflux, the direction of metabolite exchange in intact fruit will depend on the relative concentrations of each amino acid on either side of the membrane and the Km of the transporter for each amino acid. Efflux of pre‐loaded radiolabelled Glu was also *trans*‐stimulated by a number of other amino acids in mature green fruit (Figure 4c), whereas in ripe fruit the *trans*‐stimulation effect was more specific to GABA and Asp (Figure 4d), suggesting the presence of the hypothesised Glu/Asp/GABA exchanger.

We note that absolute rates of [^3^H]Glu transport tended to be lower in tonoplast vesicles isolated from red compared with green fruit, but consider these not to be directly comparable because of the greater leakiness of vesicles isolated from mature red fruit. This was apparent from the effect of the non‐ionic detergent Brij 58 on measurable ATPase activity. For green fruit tonoplast vesicles, ATPase activity was 289 ± 62 pmol P_i_ min^−1^ mg protein^−1^ in the absence of Brij 58 and 645 ± 134 P_i_ min^−1^ mg protein^−1^ in its presence. This approximately two‐fold stimulation is what is expected for well sealed vesicles with a random right‐side‐out/inside‐out orientation. In contrast, Brij 58 had negligible effect on red fruit tonoplast vesicles: ATPase activity was 257 ± 81 and 290 ± 33 pmol P_i_ min^−1^ mg protein^−1^ in the absence and presence of Brij 58, respectively. It is unclear precisely why tonoplast vesicle from red fruit should be leakier, but it could be a consequence of increased oxidative stress during the latter phases of fruit ripening (Mondal *et al*., 2004). Associated with the increased leakiness, vesicles from red fruit contained significantly less [^3^H]Glu at the end of the pre‐loading period (Figure 4a) than those from green fruit (0.076 ± 0.001 compared with 0.197 ± 0.018 μmol μg protein^−1^, respectively). Inevitably, this meant that absolute rates of [^3^H]Glu efflux measured in the *trans*‐stimulation configuration (Figure 4b) were lower in vesicles from red fruit compared with green fruit. Despite this difficulty in comparing rates of Glu transport in vesicles from fruit tissue at different developmental stages, it is notable that the specificity of the endogenous transport activity measured for Glu/Asp/GABA was actually greater in red fruit compared with green fruit (cf. Figure 4c,d), suggesting that the composition of transporters in the tonoplast membrane was altered during the course of fruit ripening.

**Figure 5:**
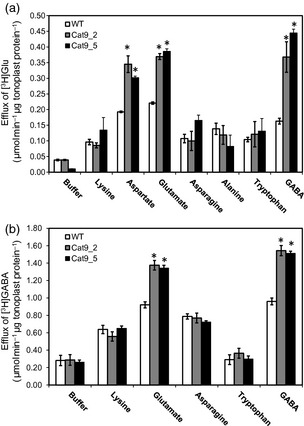
Transport rates of [^3^H]glutamate and [^3^H]GABA in the presence of different external amino acids from transgenic tomato fruit tonoplast vesicles overexpressing SlCAT9‐YFP. Tonoplast vesicles were isolated from mature green tomatoes of two independent transgenic lines (CAT 9_2 and CAT 9_5) and wild‐type (WT), pre‐loaded for 10 min with [^3^H]glutamate (a) or [^3^H]GABA, and the rate of efflux of [^3^H]glutamate (a) or [^3^H]GABA (b) measured in the presence of a range of amino acids (25 mm). A buffer control was included to test the zero‐*trans* configuration. Values are means ± standard error (SE) from three biological replicates consisting each of three technical replicates. *Indicates significantly different from WT (*t*‐test, *P* < 0.05).

To test whether the SlCAT9 protein was responsible for GABA/Glu/Asp exchange, we compared the transport properties of tonoplast vesicles isolated from transgenic and WT fruit. The mature‐green development stage was chosen to maximise the difference between WT and transgenic fruit: at this stage the endogenous SlCAT9 protein abundance is extremely low (Figure S4), while transgene levels are increasing (Figure S6). Tonoplast vesicles isolated from WT and transgenic fruit were again pre‐loaded with [^3^H]Glu, and *trans*‐stimulation of [^3^H]Glu transport by externally supplied amino acids was quantified. External amino acids were chosen to represent the different charge classes: negatively charged (Asp, Glu), positively charged (Lys), polar (Asn, Trp), uncharged (Ala) and GABA. As before, it was found that several of these amino acids *trans*‐stimulated [^3^H]Glu transport in WT tonoplast vesicles from mature green fruit, with Asp, Glu and GABA having the greatest effect (Figure 5a). Notably, [^3^H]Glu transport was significantly greater from transgenic tonoplast vesicles than WT when *trans*‐stimulated by Asp, Glu, or GABA, but was no different from WT in the presence of other external amino acids (Figure 5a). Transport of [^3^H]Glu in transgenic vesicles was also no different from WT when assayed in the absence of an external amino acid (i.e. in the zero‐*trans* configuration). This demonstrates that SlCAT9 operates as an obligate amino acid exchanger and specifically transports Glu, Asp and GABA. To confirm this, we conducted a reciprocal experiment in which tonoplast vesicles were pre‐loaded with [^3^H]GABA and the stimulation of [^3^H]GABA transport by a range of external amino acids was measured (Figure 5b). Of the amino acids tested, only GABA and Glu led to a greater degree of *trans*‐stimulation of [^3^H]GABA transport in vesicles from transgenic compared with WT fruit (note Asp was not tested).

### Effect of overexpression of SlCAT9 on fruit amino acid composition

To investigate the extent to which tonoplast transport of Asp, Glu and GABA controls their accumulation in ripening tomato fruit, the amino acid profile of ripe fruit of T2 generation transgenic plants overexpressing SlCAT9 was analysed (Table S2). It was found that overexpression of SlCAT9 led to substantial increases in the abundance of all three cognate amino acids in ripe fruit. Asp was increased five‐ to six‐fold, from 14 mmol kg fresh weight^−1^ in WT to 68 and 79 mmol kg fresh weight^−1^ in lines CAT9‐2 and CAT9‐5, respectively. Glu was increased nearly two‐fold in both transgenic lines (Glu content of 1.2, 2.0 and 2.2 mmol kg fresh weight^−1^ in WT, CAT9‐2 and CAT9‐5, respectively). GABA, which was barely detectable in WT ripe fruit, was present at levels 20‐fold higher (1.0 mmol kg fresh weight^−1^ in both transgenic lines). None of the other amino acids detectable in the ripe fruit samples was significantly altered in the transgenic lines (Table S2), in concordance with the transport measurements, suggesting that SlCAT9 specifically transports Glu, Asp and GABA. We also analysed the abundance of the principal carboxylic acids present in ripe fruit (Table S2). There were no statistically significant changes in malate or fumarate concentration, but citrate, the most abundant of the carboxylic acids, was slightly decreased in the transgenic lines (to 70–80% of wild‐type levels).

## Discussion

### Identification of a tonoplast‐localised Glu/Asp/GABA transporter

In this work, we have identified CAT9 as a tonoplast‐localised transporter that facilitates the exchange of Glu, Asp and GABA. Although proteomic studies have located several putative amino acid transporters on the tonoplast membrane (Carter *et al*., 2004; Schmidt *et al*., 2007; Whiteman *et al*., 2008), functional characterisation has been lacking and there were previously no characterised tonoplast transporters for GABA, Glu, or Asp. Transporters for these amino acids are known to exist in other plant membranes (Shelp *et al*., 2012). For example, the metabolic scheme depicted in Figure 1 requires GABA to be transported from cytosol to mitochondria where the GABA transaminases are located, and a mitochondrial GABA permease (GABP) has been identified in *Arabidopsis* (Michaeli *et al*., 2011). Additionally, GAT1 has been characterised as a high‐affinity plasma‐membrane‐localised GABA transporter in *Arabidopsis* that is specific for GABA (not transporting Glu or Asp). Transport via GAT1 is proton‐coupled (Meyer *et al*., 2006) and thus operates in a different manner to SlCAT9. The fact that SlCAT9 operates strictly in an exchange mode probably has physiological significance: the alteration in the amino acid composition of the vacuole during fruit ripening must be achieved without disturbing vacuolar turgor pressure. Achieving this via stoichiometric counter‐transport ensures that the osmolarity of the vacuole is unchanged.

### Tonoplast transport of Glu, Asp and GABA strongly influences the accumulation of these amino acids in ripe fruit

Overexpression of SlCat9 under the control of the E8 ripening promoter resulted in substantially increased accumulation in the fruit of the three cognate amino acids of the transporter. The increased accumulation of Asp and Glu is consistent with a role for the transporter in facilitating the influx of these amino acids into the vacuole in exchange for GABA efflux. However, given this scenario, it was surprising that GABA content also increased in ripe transgenic fruit by a similar amount to Glu. This may imply the presence of additional tonoplast transport systems for GABA and possibly transporters that facilitate unidirectional transport. This could lead to some unidirectional transport of GABA back into the vacuole, explaining why GABA has not completely disappeared in the ripe transgenic fruit as it does in WT fruit. This study highlights the importance of intracellular transport between vacuole and cytosol as a determinant of accumulation of metabolites in tomato fruit. The role of intracellular transporters as a control point in metabolism is well recognised (Martinoia *et al*., 2007; Linka and Weber, 2010; Sweetlove and Fernie, 2013), but is nevertheless frequently overlooked in metabolic engineering efforts. Here we demonstrate that overexpression of a single tonoplast transporter can lead to a many‐fold increase in the cognate metabolites, suggesting that considerable control of the accumulation flux is invested in this step. Note that the importance of transporters does not *per se* diminish the role of biosynthetic enzymes in the control of flux through metabolic pathways that span more than one intracellular compartment. For example, the activity of aconitase significantly affects the accumulation of citrate and malate in tomato fruit (Morgan *et al*., 2013), and overexpression of a fungal Glu dehydrogenase in tomato resulted in a two‐ to three‐fold increase in Glu content in fruits (Kisaka and Kida, 2003).

### Implications for cytosolic pH and charge balancing

GABA is recognised as an important metabolite in plants and, amongst other functions, has been suggested to play a role in control of cytosolic pH under acid load via the GABA shunt pathway, since conversion of Glu to GABA by Glu decarboxylase consumes a proton (Fait *et al*., 2008). Indeed, the relatively low cytosolic pH (~7.1) observed in tomato fruit by NMR has been linked to accumulation of GABA during the early stages of fruit development (Rolin *et al*., 2000). However, the later stages of fruit ripening are characterised by a pronounced decline in GABA content and an approximately stoichiometric increase in Glu plus Asp (Figure S1). This could be explained by SlCAT9 mediating the efflux of vacuolar GABA coupled to the influx of Glu and/or Asp, formed in the cytoplasm via operation of a ‘reverse’ GABA shunt pathway (Figure 1). This metabolite exchange across the tonoplast will have both proton‐ and charge‐balancing implications, since GABA (pI = 7.3) carries a net positive charge at the acidic pH of the vacuole, whereas Glu (pI = 3.2) and Asp (pI = 2.8) carry a net negative charge at the near‐neutral pH of the cytosol. Counterexchange of vacuolar GABA for cytosolic Glu or Asp will thus be an electrogenic process, transferring both negative charge into the vacuole and virtual protons (Ma *et al*., 2013; Tsai *et al*., 2013) into the cytosol as GABA is converted into Glu and Asp. As an obligate counterexchanger, transport across the tonoplast will be driven simply by the combined gradients of the respective substrates. But part of the driving force for this electrogenic Glu/Asp/GABA exchange system will be contributed by the tonoplast proton pumps (V‐ATPase and V‐PP_i_ase), which maintain an inside‐positive membrane potential across the tonoplast *in vivo*, and will thereby play an important role in energizing the remobilization of vacuolar GABA and accumulation of Glu and Asp in the latter stages of fruit ripening.

## Conclusions

In summary, we have established that SlCAT9 is localised to the tomato fruit tonoplast where it functions as a Glu/Asp/GABA exchanger. Moreover, overexpression of the *SlCAT9* gene leads to a substantial increase in the Asp content of ripe fruit and a lesser increase in Glu and GABA, strongly suggesting that the transport of amino acids across the vacuole is a major determinant of their accumulation during fruit development.

## Experimental Procedures

### Plant material

Tomato plants (*Solanum lycopersicum* L. cv. M82 and cv. Micro‐Tom) were grown in a controlled glasshouse under a 16 h light (24°C) and 8 h dark (21°C) regime.

### Isolation of tonoplast vesicles from tomato fruit pericarp

Tonoplast vesicles for proteomic analysis were isolated from 80 g of M82 fruit pericarp tissue using density gradient centrifugation following the procedure of Bettey and Smith (1993). The sucrose gradient consisted of 6 ml steps of 32, 24, 12 and 6% sucrose (all w/v). The membrane fraction that collected at the 6–12% sucrose interface after centrifugation at 100 000 ***g*** for 60 min was used for proteomics. ATPase activities of membrane fractions were measured in a medium based on that of Oleski *et al*. (1987) containing 50 mm Tris/MES (2‐(*N*‐morpholino)ethanesulphonic acid), pH 7.0, 50 mm KCl, 0.1 mm Na_2_EDTA, 1 mm Na_2_MoO_4_, 3 mm MgSO_4_ and 3 mm Na_2_ATP supplemented with 0.015 mg ml^−1^ Brij^®^ 58 and were assayed by the liberation of inorganic phosphate from ATP (Oleski *et al*., 1987).

### Transport assays

Tonoplast vesicles for transport assays were isolated from pericarp of 20–30 Micro‐Tom fruits. The extraction buffer was supplemented with 200 mm sodium ascorbate, and sucrose gradients consisted of 12 ml steps of 24 and 6% (w/v) sucrose. Tonoplast vesicles were collected from the 6 to 24% interface after centrifugation. Vesicles were diluted to 200 μg protein ml^−1^ in transport buffer (50 mm MES/BTP, pH 7.0, 5 mm KCl, 5 mm MgSO_4_, 1 mm DTT) and incubated with ^3^H‐labelled amino acid (l‐[2,3,4‐^3^H]glutamic acid, 1.11–2.22 TBq/mmol; [2,3‐^3^H(N)]γ‐aminobutyric acid, 0.925–2.22 TBq/mmol; both from American Radiolabelled Chemicals Inc., Royston, UK, http://www.arc-inc.com/) at 0.037 MBq/20 μg tonoplast protein at 25°C. At the required time points, 100 μl aliquots were vacuum‐filtered onto nitrocellulose filters (Millipore, Watford, UK, www.emdmillipore.com) and the filters immediately washed with 10 ml of ice‐cold transport buffer. Each filter was then dissolved overnight in 4 ml of HiSafe3 liquid scintillant (Thermo Fisher Scientific, Loughborough, UK, www.thermofisher.com/) before quantification of the radioactivity by scintillation counting. To assess efflux rates, vesicles were pre‐loaded with ^3^H‐labelled amino acid (0.037 MBq/20 μg protein) at 25°C for 10 min prior to 100‐fold dilution in transport buffer or transport buffer supplemented with 25 mm unlabelled amino acid. At the required time points, 1 ml samples were vacuum‐filtered, washed and dissolved for counting as above.

### Antibody production

The anti‐CAT9 antibody was raised in rabbits against the synthetic peptide SSALRSKPLASPSET as a service by Eurogentec Ltd (Fawley, UK, www.eurogentec.com/).

### Quantitative proteomic analysis of tonoplast membranes

For proteomic analysis, tryptic peptides were generated from 15 μg of carbonate‐washed tonoplast‐enriched membrane samples. Samples were fractionated on an Ultimate 3000 RSLCnano HPLC (Dionex, Camberley, UK, www.dionex.com/) system run in direct injection mode coupled to a LTQ XL Orbitrap mass spectrometer (Thermo Electron, Hemel Hempstead, UK, www.thermoscientific.com/). Samples were resolved on a 15 cm × 75 μm inner diameter picotip analytical column (New Objective, Woburn, MA, USA, www.newobjective.com/) which was packed in‐house with ProntoSIL 120‐3 C18 Ace‐EPS phase, 3 μm bead size (Bischoff Chromatography, Leonberg, Germany, www.bischoff-chrom.com/). Precursor scans were performed in the Orbitrap at a resolving power of 60 000, from which the five most intense precursor ions were selected and fragmented in the linear ion trap using CID at a normalised collision energy of 35%. Charge state +1 ions were rejected from isolation for fragmentation. Dynamic exclusion was enabled for 40 sec. Data were converted from RAW to MGF using ProteoWizard (Kessner *et al*., 2008). Data analysis and label‐free quantitation was performed using Progenesis LCMS (Nonlinear Dynamics, Newcastle, UK, www.nonlinear.com/) and data were searched using Mascot (Matrix Science, London, UK, www.matrixscience.com/) against a protein database constructed from build 1.0 of the *Solanum lycopersicum* genome (The Tomato Genome Consortium, 2012).

### Recombinant DNA manipulations

The *E. coli* MC1061 strain was used for amplification of all plasmids. All restriction enzyme were purchased from New England BioLabs (Hitchin, UK, https://www.neb.com/) and *Taq* polymerase Phusion (Thermo Fisher Scientific Ltd) was used for all PCR amplifications. cDNA was transcribed using Fermentas RevertAid Reverse Transcriptase (Thermo Fisher Scientific Ltd) starting from 1 μg of fruit pericarp RNA [extracted using the cetyltrimethylammonium bromide method (Chang *et al*., 1993)] and using the oligodT primer (5′‐TTTTTTTTTTTTTTTTTTTTV‐3′). The SlCAT9 coding region was amplified from the resulting cDNA using the following primer pair: SlCAT9s (5′‐AGAATCCGATCGATGAGTACTTGGGAACGC‐3′) and SlCAT9as (5′‐ GTAAGAACTCTAGATTAATCAGCTTCTGTAGG‐3′). The PCR product was digested with *Cla*I and *Xba*I and cloned behind the cauliflower mosaic virus 35S promoter in the plasmid Amy‐spo (Pimpl *et al*., 2003). The resulting plasmid was named p35S‐SlCAT9. To generate the SlCAT9‐YFP fusion, SlCAT9 was amplified from the p35S‐SlCAT9 vector using the primer pair SlCAT9s and SlCAT9K (5′‐GTAAGAACGGTACCATCAGCTTCTGTAGGTGC‐3′) resulting in a PCR product lacking a stop codon. This PCR product was digested with *Cla*I and *Kpn*I and a YFP DNA fragment with *Kpn*I and *Xba*I. Both digested products were ligated into the vector Amy‐spo to generate the plasmid p35S‐SlCAT9‐YFP. For plant transformation and *Agrobacterium*‐mediated transient expression, the 35S‐SlCAT9‐YFP‐3′ nos cassette was subcloned as an *Eco*RI–*Hin*dIII fragment into the T‐DNA vector pDE1001 (Denecke *et al*., 1993). The resulting plasmid was named pT‐35S‐SlCAT9‐YFP. The E8 promoter was amplified from tomato genomic DNA using the following oligo pair E8s (5′‐TACCGTGCGAATTCTCCCTAATGATATTGTTCATGTA‐3′) and E8as (5′‐TTCGACCATCGATTCTTTTGCACTGTGAATGATT‐3′). The resulting PCR product was cloned into the vector pT‐35S‐SlCAT9‐YFP using the restriction sites *Eco*RI and *Cla*I to remove the 35S promoter and replace it with the E8 promoter. This plasmid was named pT‐E8‐SlCAT9‐YFP.

### Confocal laser scanning microscopy

Confocal imaging was performed using a Zeiss LSM 510 META laser scanning microscope (www.zeiss.co.uk/) with a Plan‐Neofluar 25×/0.8 Imm Corr DIC objective. For imaging of YFP fusions, the 514 nm excitation line of an argon ion laser was used. Fluorescence was detected using a 458/514‐nm dichroic beam splitter and a 535‐ to 590‐nm band‐pass filter. Post‐acquisition image processing was performed with the LSM 5 image browser (Zeiss, Oberkochen, Germany).

### Plant transformation

Tobacco plants (*Nicotiana tabacum* L. ‘Petit Havana’ SR1) were infiltrated with cultures (at an optical density of 0.1) of *Agrobacterium tumefaciens* (GV3101), harbouring the vector pT‐35S‐SlCAT9‐YFP, as described previously (Neuhaus and Boevink, 2001) and analysed after 3 days of further growth. Generation of stably‐transformed tomato lines was via *Agrobacterium*‐mediated transformation of cotyledons (Van Eck *et al*., 2006).

### Metabolite analysis

Metabolite analysis was by gas chromatography mass spectrometry of chloroform–methanol extracts of tomato pericarp derivatised with *N*‐methyl‐*N*‐(trimethylsilyl)trifluoroacetamide (Lisec *et al*., 2006).

## Supporting information


**Data S1.** Shotgun proteomics of tonoplast‐enriched membrane fractions from tomato fruit.

**Table S1.** Integral membrane tonoplast proteins identified by proteomic analysis of isolated tonoplast‐enriched membrane fractions from tomato fruit.

**Table S2.** Metabolite content of ripe transgenic tomato fruit overexpressing SlCAT9.

**Figure S1.** Changes of key acidic metabolites during fruit development.

**Figure S2.** Assessment of purity of tonoplast membrane fractions.

**Figure S3.** Phylogenetic analysis of selected plant CAT proteins.

**Figure S4.** Quantification and localisation of SlCat9 by western blotting.

**Figure S5.** Characterisation of transgenic tomato plants expressing SlCat9YFP under the control of the ethylene‐inducible E8 promoter.

**Figure S6.** Quantification of SlCAT9‐YFP transgene expression during fruit development by YFP fluorescence.

## References

[tpj12766-bib-0001] Akihiro, T. , Koike, S. , Tani, R. ***et al.*** (2008) Biochemical mechanism on GABA accumulation during fruit development in tomato. Plant Cell Physiol. 49, 1378–1389. 18713763 10.1093/pcp/pcn113

[tpj12766-bib-0002] Bettey, M. and Smith, J.A. (1993) Dicarboxylate transport at the vacuolar membrane of the CAM plant *Kalanchoe daigremontiana*: sensitivity to protein‐modifying and sulphydryl reagents. Biochim. Biophys. Acta, 1152, 270–279. 8218327 10.1016/0005-2736(93)90258-2

[tpj12766-bib-0003] Carrari, F. , Baxter, C. , Usadel, B. ***et al.*** (2006) Integrated analysis of metabolite and transcript levels reveals the metabolic shifts that underlie tomato fruit development and highlight regulatory aspects of metabolic network behavior. Plant Physiol. 142, 1380–1396. 17071647 10.1104/pp.106.088534 PMC1676044

[tpj12766-bib-0004] Carter, C. , Pan, S. , Zouhar, J. , Avila, E.L. , Girke, T. and Raikhel, N.V. (2004) The vegetative vacuole proteome of *Arabidopsis thaliana reveals* predicted and unexpected proteins. Plant Cell, 16, 3285–3303. 15539469 10.1105/tpc.104.027078 PMC535874

[tpj12766-bib-0048] Chang, S. , Puryear, J. and Cairney, J. (1993) A simple and effcient method for isolating RNA from pine trees. Plan Mol Biol Rep., 11, 113–116.

[tpj12766-bib-0006] Deikman, J. and Fischer, R.L. (1988) Interaction of a DNA binding factor with the 5′‐flanking region of an ethylene‐responsive fruit ripening gene from tomato. EMBO J. 7, 3315–3320. 3208738 10.1002/j.1460-2075.1988.tb03202.x PMC454826

[tpj12766-bib-0049] Denecke, J. , Ek, B. , Caspers, M. , Sinjorgo, K.M.C. and Palva, E.T. (1993) Analysis of sorting signals responsible for the accumulation of soluble reticuloplasmins in the plant endoplasmic reticulum. J Exp Bot., 44, 213–221.

[tpj12766-bib-0007] Endler, A. , Meyer, S. , Schelbert, S. , Schneider, T. , Weschke, W. , Peters, S.W. , Keller, F. , Baginsky, S. , Martinoia, E. and Schmidt, U.G. (2006) Identification of a vacuolar sucrose transporter in barley and Arabidopsis mesophyll cells by a tonoplast proteomic approach. Plant Physiol. 141, 196–207. 16581873 10.1104/pp.106.079533 PMC1459324

[tpj12766-bib-0008] Eshed, Y. and Zamir, D. (1995) An introgression line population of *Lycopersicon pennellii* in the cultivated tomato enables the identification and fine mapping of yield‐associated QTL. Genetics, 141, 1147–1162. 8582620 10.1093/genetics/141.3.1147 PMC1206837

[tpj12766-bib-0009] Fait, A. , Fromm, H. , Walter, D. , Galili, G. and Fernie, A.R. (2008) Highway or byway: the metabolic role of the GABA shunt in plants. Trends Plant Sci. 13, 14–19. 18155636 10.1016/j.tplants.2007.10.005

[tpj12766-bib-0010] Jaquinod, M. , Villiers, F. , Kieffer‐Jaquinod, S. , Hugouvieux, V. , Bruley, C. , Garin, J. and Bourguignon, J. (2007) A proteomics dissection of *Arabidopsis thalian*a vacuoles isolated from cell culture. Mol. Cell Proteomics, 6, 394–412. 17151019 10.1074/mcp.M600250-MCP200 PMC2391258

[tpj12766-bib-0011] Johnson, C. , Hall, J.L. and Ho, L.C. (1988) Pathways of uptake and accumulation of sugars in tomato fruit. Ann. Bot. 61, 593–603.

[tpj12766-bib-0012] Kessner, D. , Chambers, M. , Burke, R. , Agus, D. and Mallick, P. (2008) ProteoWizard: open source software for rapid proteomics tools development. Bioinformatics, 24, 2534–2536. 18606607 10.1093/bioinformatics/btn323 PMC2732273

[tpj12766-bib-0013] Kisaka, H. and Kida, T. (2003) Transgenic tomato plant carrying a gene for NADP‐dependent glutamate dehydrogenase (gdhA) from *Aspergillus nidulans* . Plant Sci. 164, 35–42.

[tpj12766-bib-0014] Klee, H.J. and Giovannoni, J.J. (2011) Genetics and control of tomato fruit ripening and quality attributes. Annu. Rev. Genet. 45, 41–59. 22060040 10.1146/annurev-genet-110410-132507

[tpj12766-bib-0015] Koike, S. , Matsukura, C. , Takayama, M. , Asamizu, E. and Ezura, H. (2013) Suppression of gamma‐aminobutyric acid (GABA) transaminases induces prominent GABA accumulation, dwarfism and infertility in the tomato (*Solanum lycopersicum* L.). Plant Cell Physiol. 54, 793–807. 23435575 10.1093/pcp/pct035

[tpj12766-bib-0016] Linka, N. and Weber, A.P. (2010) Intracellular metabolite transporters in plants. Mol. Plant, 3, 21–53. 20038549 10.1093/mp/ssp108

[tpj12766-bib-0017] Lisec, J. , Schauer, N. , Kopka, J. , Willmitzer, L. and Fernie, A.R. (2006) Gas chromatography mass spectrometry‐based metabolite profiling in plants. Nat. Protoc. 1, 387–395. 17406261 10.1038/nprot.2006.59

[tpj12766-bib-0018] Ma, D. , Lu, P. , Yan, C. , Fan, C. , Yin, P. , Wang, J. and Shi, Y. (2012) Structure and mechanism of a glutamate‐GABA antiporter. Nature, 483, 632–636. 22407317 10.1038/nature10917

[tpj12766-bib-0019] Ma, D. , Lu, P. and Shi, Y. (2013) Substrate selectivity of the acid‐activated glutamate/gamma‐aminobutyric acid (GABA) antiporter GadC from *Escherichia coli* . J. Biol. Chem. 288, 15148–15153. 23589309 10.1074/jbc.M113.474502 PMC3663535

[tpj12766-bib-0020] Martinoia, E. , Maeshima, M. and Neuhaus, H.E. (2007) Vacuolar transporters and their essential role in plant metabolism. J. Exp. Bot. 58, 83–102. 17110589 10.1093/jxb/erl183

[tpj12766-bib-0021] Martinoia, E. , Meyer, S. , De Angeli, A. and Nagy, R. (2012) Vacuolar transporters in their physiological context. Annu. Rev. Plant Biol. 63, 183–213. 22404463 10.1146/annurev-arplant-042811-105608

[tpj12766-bib-0022] Meyer, A. , Eskandari, S. , Grallath, S. and Rentsch, D. (2006) AtGAT1, a high affinity transporter for gamma‐aminobutyric acid in *Arabidopsis thaliana* . J. Biol. Chem. 281, 7197–7204. 16407306 10.1074/jbc.M510766200 PMC3009663

[tpj12766-bib-0023] Michaeli, S. , Fait, A. , Lagor, K. ***et al.*** (2011) A mitochondrial GABA permease connects the GABA shunt and the TCA cycle, and is essential for normal carbon metabolism. Plant J. 67, 485–498. 21501262 10.1111/j.1365-313X.2011.04612.x

[tpj12766-bib-0024] Mondal, K. , Sharma, N.S. , Malhotra, S.P. , Dhawan, K. and Singh, R. (2004) Antioxidant systems in ripening tomato fruits. Biol. Plant, 48, 49–53.

[tpj12766-bib-0025] Morgan, M.J. , Osorio, S. , Gehl, B. , Baxter, C.J. , Kruger, N.J. , Ratcliffe, R.G. , Fernie, A.R. and Sweetlove, L.J. (2013) Metabolic engineering of tomato fruit organic acid content guided by biochemical analysis of an introgression line. Plant Physiol. 161, 397–407. 23166354 10.1104/pp.112.209619 PMC3532270

[tpj12766-bib-0026] Neuhaus, J.M. and Boevink, P. (2001) The green fluorescent protein (GFP) as a reporter in plant cells In Plant Cell Biology: A Practical Approach (Hawes C. and Satiat‐Jenunemaitre B., eds). Oxford, UK: Oxford University Press, pp. 127–142.

[tpj12766-bib-0027] Okumoto, S. and Pilot, G. (2011) Amino acid export in plants: a missing link in nitrogen cycling. Mol. Plant, 4, 453–463. 21324969 10.1093/mp/ssr003 PMC3143828

[tpj12766-bib-0028] Oleski, N. , Mahdavi, P. , Peiser, G. and Bennett, A.B. (1987) Transport properties of the tomato fruit tonoplast: I. Identification and characterization of an anion‐sensitive H‐ATPase. Plant Physiol. 84, 993–996. 16665635 10.1104/pp.84.4.993 PMC1056715

[tpj12766-bib-0029] Overy, S.A. , Walker, H.J. , Malone, S. , Howard, T.P. , Baxter, C.J. , Sweetlove, L.J. , Hill, S.A. and Quick, W.P. (2005) Application of metabolite profiling to the identification of traits in a population of tomato introgression lines. J. Exp. Bot. 56, 287–296. 15596481 10.1093/jxb/eri070

[tpj12766-bib-0050] Palmieri, F. , Pierri, C.L. , De Grassi, A. , Nunes‐Nesi, A. and Fernie, A.R. (2011) Evolution, structure and function of mitochondrial carriers: a review with new insights. Plant J. 66, 161–181. 21443630 10.1111/j.1365-313X.2011.04516.x

[tpj12766-bib-0051] Pimpl, P. , Hanton, S.L. , Taylor, J.P. , Pinto‐daSilva, L.L. and Denecke, J. (2003) The GTPase ARF1p controls the sequence‐specific vacuolar sorting route to the lytic vacuole. Plant Cell, 15, 1242–1256. 12724547 10.1105/tpc.010140 PMC153729

[tpj12766-bib-0030] Rolin, D. , Baldet, P. , Just, D. , Chevalier, C. , Biran, M. and Raymond, P. (2000) NMR study of low subcellular pH during the development of cherry tomato fruit. Aust. J. Plant Physiol. 27, 61–69.

[tpj12766-bib-0031] Saier, M.H. Jr , Yen, M.R. , Noto, K. , Tamang, D.G. and Elkan, C. (2009) The transporter classification database: recent advances. Nucleic Acids Res. 37, D274–D278. 19022853 10.1093/nar/gkn862 PMC2686586

[tpj12766-bib-0032] Schauer, N. , Semel, Y. , Roessner, U. ***et al.*** (2006) Comprehensive metabolic profiling and phenotyping of interspecific introgression lines for tomato improvement. Nat. Biotechnol. 24, 447–454. 16531992 10.1038/nbt1192

[tpj12766-bib-0033] Schmidt, U.G. , Endler, A. , Schelbert, S. , Brunner, A. , Schnell, M. , Neuhaus, H.E. , Marty‐Mazars, D. , Marty, F. , Baginsky, S. and Martinoia, E. (2007) Novel tonoplast transporters identified using a proteomic approach with vacuoles isolated from cauliflower buds. Plant Physiol. 145, 216–229. 17660356 10.1104/pp.107.096917 PMC1976570

[tpj12766-bib-0034] Shelp, B.J. , Mullen, R.T. and Waller, J.C. (2012) Compartmentation of GABA metabolism raises intriguing questions. Trends Plant Sci. 17, 57–59. 22226724 10.1016/j.tplants.2011.12.006

[tpj12766-bib-0035] Shiratake, K. and Martinoia, E. (2007) Transporters in fruit vacuoles. Plant Biotechnol. 24, 127–133.

[tpj12766-bib-0036] Sorrequieta, A. , Ferraro, G. , Boggio, S.B. and Valle, E.M. (2010) Free amino acid production during tomato fruit ripening: a focus on L‐glutamate. Amino Acids, 38, 1523–1532. 19876714 10.1007/s00726-009-0373-1

[tpj12766-bib-0037] Stein, W.D. (1990) Channels, Carriers, and Pumps: An Introduction to Membrane transport. San Diego, CA: Academic Press.

[tpj12766-bib-0038] Su, Y.H. , Frommer, W.B. and Ludewig, U. (2004) Molecular and functional characterization of a family of amino acid transporters from Arabidopsis. Plant Physiol. 136, 3104–3113. 15377779 10.1104/pp.104.045278 PMC523371

[tpj12766-bib-0039] Sweetlove, L.J. and Fernie, A.R. (2013) The spatial organization of metabolism within the plant cell. Annu. Rev. Plant Biol. 64, 723–746. 23330793 10.1146/annurev-arplant-050312-120233

[tpj12766-bib-0040] Tegeder, M. (2014) Transporters involved in source to sink partitioning of amino acids and ureides: opportunities for crop improvement. J. Exp. Bot. 65, 1865–1878. 24489071 10.1093/jxb/eru012

[tpj12766-bib-0052] The Tomato Genome Consortium (2012) The tomato genome sequence provides insights into fleshy fruit evolution. Nature, 485, 635–641. 22660326 10.1038/nature11119 PMC3378239

[tpj12766-bib-0041] Tsai, M.F. , McCarthy, P. and Miller, C. (2013) Substrate selectivity in glutamate‐dependent acid resistance in enteric bacteria. Proc. Natl Acad. Sci. USA, 110, 5898–5902. 23530225 10.1073/pnas.1301444110 PMC3625338

[tpj12766-bib-0042] Tusnady, G.E. and Simon, I. (2001) The HMMTOP transmembrane topology prediction server. Bioinformatics, 17, 849–850. 11590105 10.1093/bioinformatics/17.9.849

[tpj12766-bib-0043] Van Eck, J. , Kirk, D.D. and Walmsley, A.M. (2006) Tomato (*Lycopersicum esculentum*) In Agrobacterium Protocols (Wang K. ed.). New York, NY: Humana Press, pp. 459–474. 10.1385/1-59745-130-4:459 16988368

[tpj12766-bib-0044] Whiteman, S.A. , Serazetdinova, L. , Jones, A.M. , Sanders, D. , Rathjen, J. , Peck, S.C. and Maathuis, F.J. (2008) Identification of novel proteins and phosphorylation sites in a tonoplast enriched membrane fraction of *Arabidopsis thaliana* . Proteomics, 8, 3536–3547. 18686298 10.1002/pmic.200701104

[tpj12766-bib-0045] Wong, F.H. , Chen, J.S. , Reddy, V. , Day, J.L. , Shlykov, M.A. , Wakabayashi, S.T. and Saier, M.H. Jr (2012) The amino acid‐polyamine‐organocation superfamily. J. Mol. Microbiol. Biotechnol. 22, 105–113. 22627175 10.1159/000338542

[tpj12766-bib-0046] Yang, H. , Bogner, M. , Stierhof, Y.D. and Ludewig, U. (2010) H‐independent glutamine transport in plant root tips. PLoS ONE, 5, e8917. 20111724 10.1371/journal.pone.0008917 PMC2811748

[tpj12766-bib-0047] Yin, Y.G. , Tominaga, T. , Iijima, Y. , Aoki, K. , Shibata, D. , Ashihara, H. , Nishimura, S. , Ezura, H. and Matsukura, C. (2010) Metabolic alterations in organic acids and gamma‐aminobutyric acid in developing tomato (*Solanum lycopersicum* L.) fruits. Plant Cell Physiol. 51, 1300–1314. 20595461 10.1093/pcp/pcq090

