# Supplementary material for: A tonoplast Glu/Asp/GABA exchanger that affects tomato fruit amino acid composition
Source: Plant J. 2015 Feb 24;81(5):651–60. doi: 10.1111/tpj.12766 (PMC4950293; doi:10.1111/tpj.12766)
Supplement: Supplementary file 2 — Table S1. Integral membrane tonoplast proteins identified by proteomic analysis of isolated tonoplast‐enriched membrane fractions from tomato fruit. [file TPJ-81-651-s002.docx]

**Supporting Information Table S1**

| Protein name | Accession number |  |
| --- | --- | --- |
|  |  |  |
|  | SL1.00 genome  (ITAG release 1.00) | SL2.50 genome  (ITAG release 2.40) |
| **V-ATPase subunits and pyrophosphatases** |  |  |
| V-type proton ATPase subunit 116 kDa subunit a isoform 4 | SL1.00sc05477_124.1.1 | Solyc01g110120.2.1 |
| Vacuolar proton pyrophosphatase | SL1.00sc04007_110.1.1 | Solyc03g117480.2.1 |
| **Aquaporins** |  |  |
| Tonoplast intrinsic protein 2 water channel  (Aquaporin-like protein IPR012269) | SL1.00sc06019_181.1.1 | Solyc06g074820.2.1 |
| **Amino-acid transporters** |  |  |
| Cationic amino acid transporter  (Amino acid transporter IPR015606) | SL1.00sc04801_50.1.1 | Solyc10g018600.1.1 |
| Amino acid transporter family protein  (Amino acid transporter IPR013057) | SL1.00sc07184_335.1.1 | Solyc11g008440.1.1 |
| **Metal transporters** |  |  |
| NRAMP4 transmembrane transporter | SL1.00sc04604_10.1.1 | Solyc02g092800.2.1 |
| COPT5 copper ion transmembrane transporter | SL1.00sc00226_751.1.1 | Solyc02g082080.1.1 |
| **Carbohydrate transporters** |  |  |
| Sugar transporter ERD6-like 6  (IPR003663 Sugar/inositol transporter) | SL1.00sc02102_420.1.1 | Solyc12g089180.1.1 |
| Sugar transporter ERD6-like 6  (IPR003663 Sugar/inositol transporter) | SL1.00sc02164_311.1.1 | Solyc04g080460.2.1 |
| Sugar transporter ERD6-like 5  (IPR003663 Sugar/inositol transporter) | SL1.00sc05858_579.1.1 | Solyc01g098560.2.1 |
| D-xylose-proton symporter-like 2  (IPR003663 Sugar/inositol transporter) | SL1.00sc04889_97.1.1 | Solyc03g096950.2.1 |
| TMT2 tonoplast monosaccharide transporter 2  (IPR016196 Major facilitator superfamily) | SL1.00sc02164_525.1.1 | Solyc04g082700.2.1 |
| TMT2tonoplast monosaccharide transporter 2  (IPR016196 Major facilitator superfamily) | SL1.00sc06741_63.1.1 | Solyc03g032040.2.1 |
|  |  |  |
| **Ion channels and transporters** |  |  |
| Potassium transporter 7  (IPR003855 K^+^ potassium transporter) | SL1.00sc06818_194.1.1 | Solyc10g047270.1.1 |
| CLC-C chloride channel C | SL1.00sc05858_921.1.1 | Solyc01g103140.2.1 |
| **Other transporters** |  |  |
| Multidrug resistance protein ABC transporter family  (IPR001140 ABC transporter, transmembrane region) | SL1.00sc05189_129.1.1 | Solyc01g080640.2.1 |
| Multidrug resistance protein ABC transporter family  (IPR003439 ABC transporter-like) | SL1.00sc03179_170.1.1 | Solyc12g044820.1.1 |
| Multidrug resistance protein ABC transporter family  (IPR001140 ABC transporter, transmembrane region) | SL1.00sc05382_104.1.1 | Solyc09g075020.2.1 |
| Multidrug resistance protein ABC transporter family  (IPR001140 ABC transporter, transmembrane region) | SL1.00sc05189_128.1.1 | Solyc01g080640.2.1 |
| Peptide transporter PTR2  (Peptide transporter IPR000109) | SL1.00sc01651_25.1.1 | Solyc02g021240.2.1 |
| Nucleobase-ascorbate transporter 3  (IPR006043 Xanthine/uracil/vitamin C permease) | SL1.00sc02164_211.1.1 | Solyc04g079430.2.1 |
