# Supplementary material for: A tonoplast Glu/Asp/GABA exchanger that affects tomato fruit amino acid composition
Source: Plant J. 2015 Feb 24;81(5):651–60. doi: 10.1111/tpj.12766 (PMC4950293; doi:10.1111/tpj.12766)
Supplement: Supplementary file 3 — Table S2. Metabolite content of ripe transgenic tomato fruit overexpressing SlCAT9. [file TPJ-81-651-s003.docx]

**Supplemental Table S2.** Metabolite content of ripe transgenic tomato fruit overexpressing SlCAT9.

Ripe fruit (harvested at 38 days after anthesis) from wild type and T_2_-generation transgenic Micro=Tom plants were harvested and pericarp samples taken. For each sample, 4 fruits were pooled from a single plant. Values shown are the means ± SE of 6 samples, each taken from an independent plant. WT, wild type; nd, not detectable. Values in bold are significantly different from WT (*t*-test; P < 0.05).

|  | **Metabolite content (mmol/kg fresh weight)** | | |
| --- | --- | --- | --- |
| **Metabolite** |  |  |  |
|  | **WT** | **CAT9_2** | **CAT9_5** |
| ***Amino acids*** |  |  |  |
| Aspartate | 13.7±2.8 | **68.4±2.7** | **79.1±4.5** |
| Glutamate | 1.18±0.18 | **1.99±0.11** | **2.18±0.15** |
| Arginine | 0.15±0.001 | 0.15±0.002 | 0.15±0.002 |
| Asparagine | 0.28±0.01 | 0.45±0.05 | 0.49±0.07 |
| Serine | 0.05±0.05 | nd | nd |
| Cysteine | 0.15±0.02 | 0.12±0.04 | 0.30±0.03 |
| Glycine | 1.81±0.01 | 1.72±0.02 | 1.78±0.03 |
| Proline | nd | nd | 0.04±0.03 |
| Alanine | 0.40±0.17 | nd | nd |
| Isoleucine | 0.03±0.01 | 0.07±0.01 | 0.07±0.01 |
| Phenylalanine | 0.12±0.02 | 0.18±0.01 | 0.22±0.02 |
| Tryptophan | 0.06±0.001 | 0.06±0.001 | 0.07±0.001 |
| Tyrosine | 0.05±0.01 | nd | nd |
| GABA | 0.05±0.05 | **0.98±0.07** | **1.02±0.13** |
| Lysine | 0.31±0.12 | 0.28±0.08 | 0.45±0.11 |
| ***Carboxylic acids*** |  |  |  |
| Citrate | 16.6±0.3 | **13.7±0.4** | **11.5±0.2** |
| Fumarate | 0.21±0.002 | 0.20±0.002 | 0.21±0.002 |
| Malate | 1.52±0.04 | 1.45±0.09 | 1.02±0.03 |
|  |  |  |  |
