# Supplementary material for: A tonoplast Glu/Asp/GABA exchanger that affects tomato fruit amino acid composition
Source: Plant J. 2015 Feb 24;81(5):651–60. doi: 10.1111/tpj.12766 (PMC4950293; doi:10.1111/tpj.12766)
Supplement: Supplementary file 4 — Figure S1. Changes of key acidic metabolites during fruit development. [file TPJ-81-651-s004.pptx]

## Slide 1
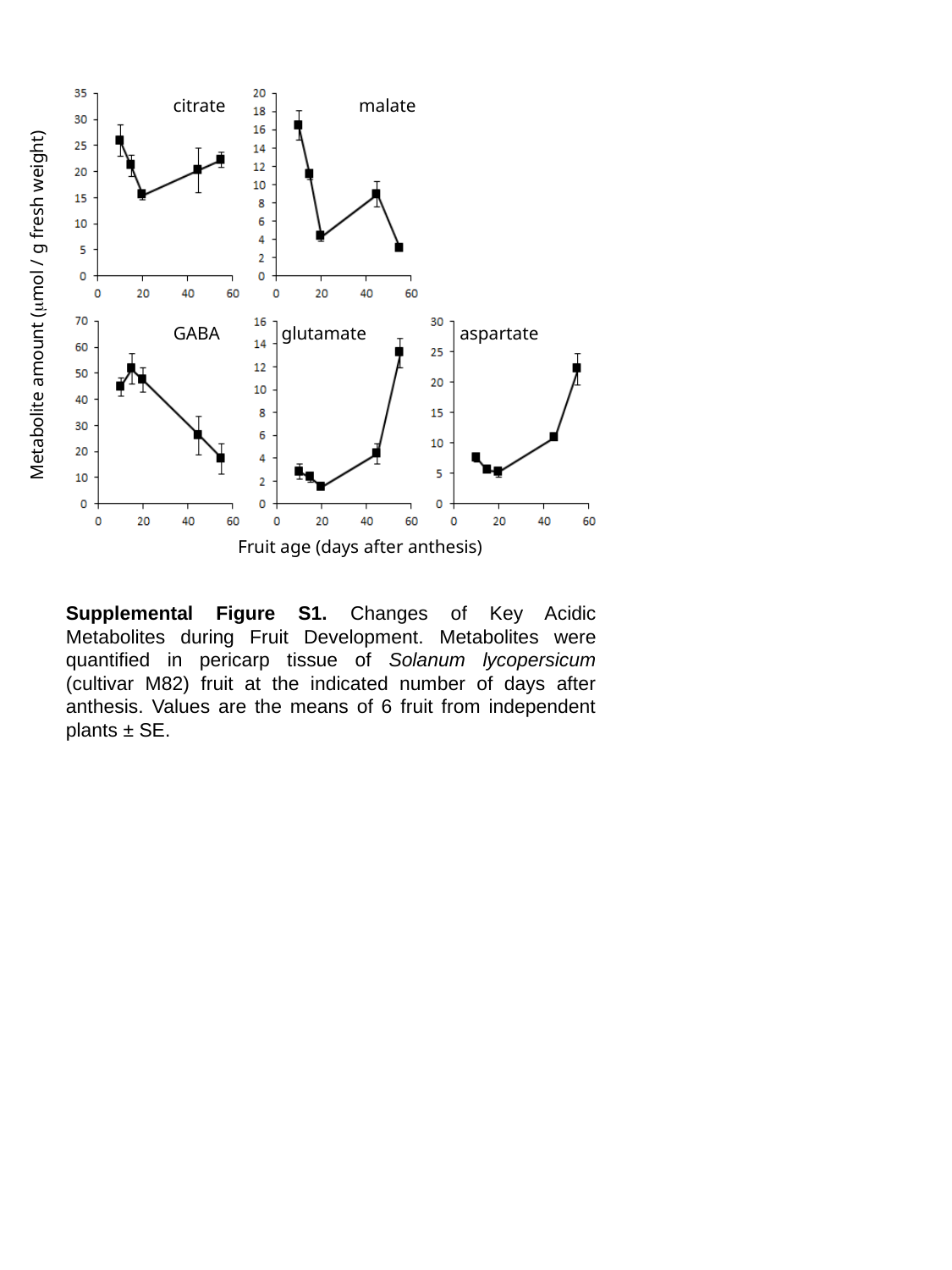

malate
citrate
Metabolite amount (mmol / g fresh weight)
GABA
glutamate
aspartate
Fruit age (days after anthesis)
Supplemental Figure S1. Changes of Key Acidic Metabolites during Fruit Development. Metabolites were quantified in pericarp tissue of Solanum lycopersicum (cultivar M82) fruit at the indicated number of days after anthesis. Values are the means of 6 fruit from independent plants ± SE.
