# Supplementary material for: A tonoplast Glu/Asp/GABA exchanger that affects tomato fruit amino acid composition
Source: Plant J. 2015 Feb 24;81(5):651–60. doi: 10.1111/tpj.12766 (PMC4950293; doi:10.1111/tpj.12766)
Supplement: Supplementary file 5 — Figure S2. Assessment of purity of tonoplast membrane fractions. [file TPJ-81-651-s005.pptx]

## Slide 1
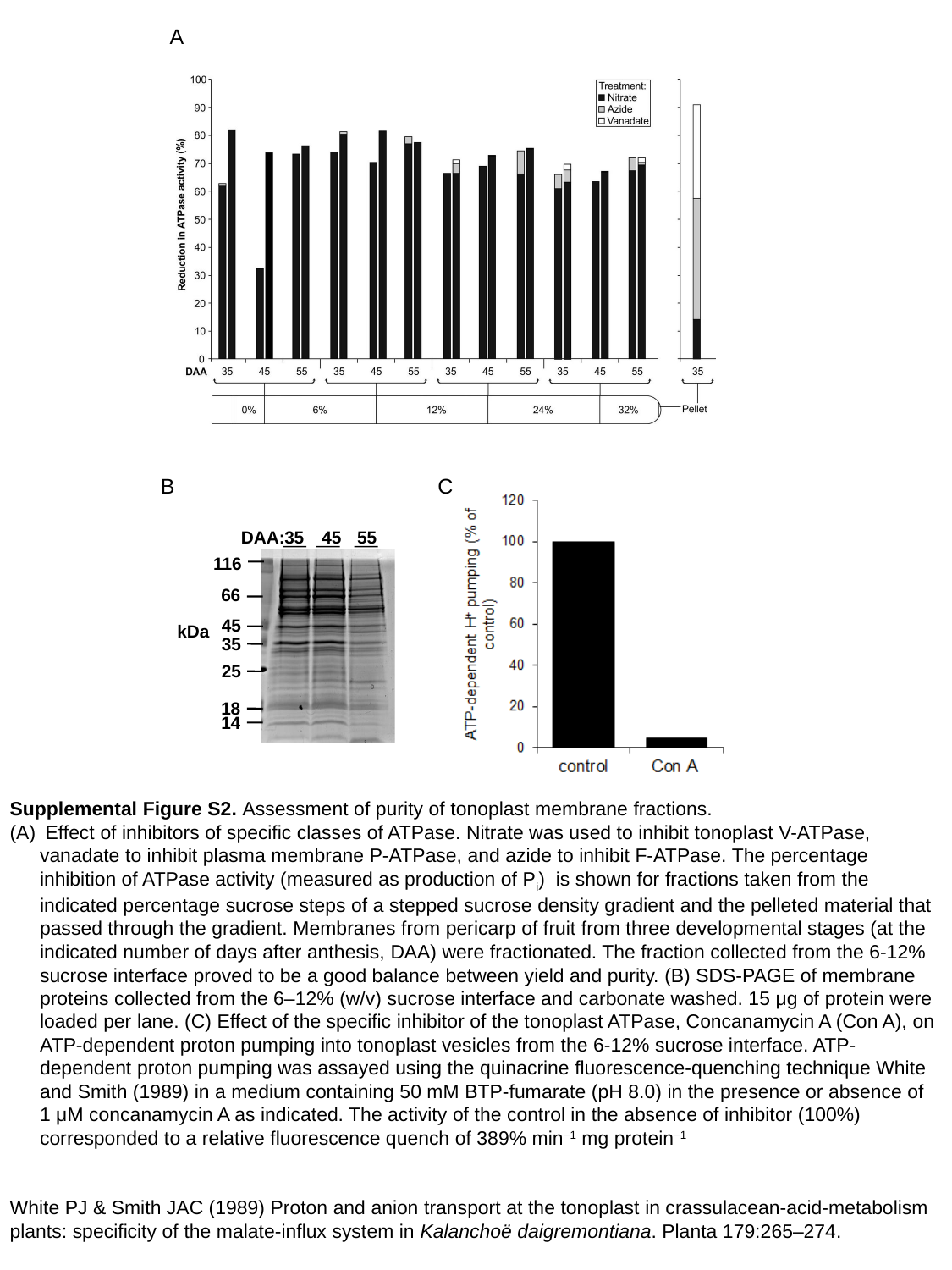

A
B
C
DAA:
35
45
55
116
66
45
kDa
35
25
18
14
Supplemental Figure S2. Assessment of purity of tonoplast membrane fractions.
 Effect of inhibitors of specific classes of ATPase. Nitrate was used to inhibit tonoplast V-ATPase, vanadate to inhibit plasma membrane P-ATPase, and azide to inhibit F-ATPase. The percentage inhibition of ATPase activity (measured as production of Pi) is shown for fractions taken from the indicated percentage sucrose steps of a stepped sucrose density gradient and the pelleted material that passed through the gradient. Membranes from pericarp of fruit from three developmental stages (at the indicated number of days after anthesis, DAA) were fractionated. The fraction collected from the 6-12% sucrose interface proved to be a good balance between yield and purity. (B) SDS-PAGE of membrane proteins collected from the 6–12% (w/v) sucrose interface and carbonate washed. 15 μg of protein were loaded per lane. (C) Effect of the specific inhibitor of the tonoplast ATPase, Concanamycin A (Con A), on ATP-dependent proton pumping into tonoplast vesicles from the 6-12% sucrose interface. ATP-dependent proton pumping was assayed using the quinacrine fluorescence-quenching technique White and Smith (1989) in a medium containing 50 mM BTP-fumarate (pH 8.0) in the presence or absence of 1 μM concanamycin A as indicated. The activity of the control in the absence of inhibitor (100%) corresponded to a relative fluorescence quench of 389% min−1 mg protein−1
White PJ & Smith JAC (1989) Proton and anion transport at the tonoplast in crassulacean-acid-metabolism plants: specificity of the malate-influx system in Kalanchoë daigremontiana. Planta 179:265–274.
