# Supplementary material for: A tonoplast Glu/Asp/GABA exchanger that affects tomato fruit amino acid composition
Source: Plant J. 2015 Feb 24;81(5):651–60. doi: 10.1111/tpj.12766 (PMC4950293; doi:10.1111/tpj.12766)
Supplement: Supplementary file 6 — Figure S3. Phylogenetic analysis of selected plant CAT proteins. [file TPJ-81-651-s006.pptx]

## Slide 1
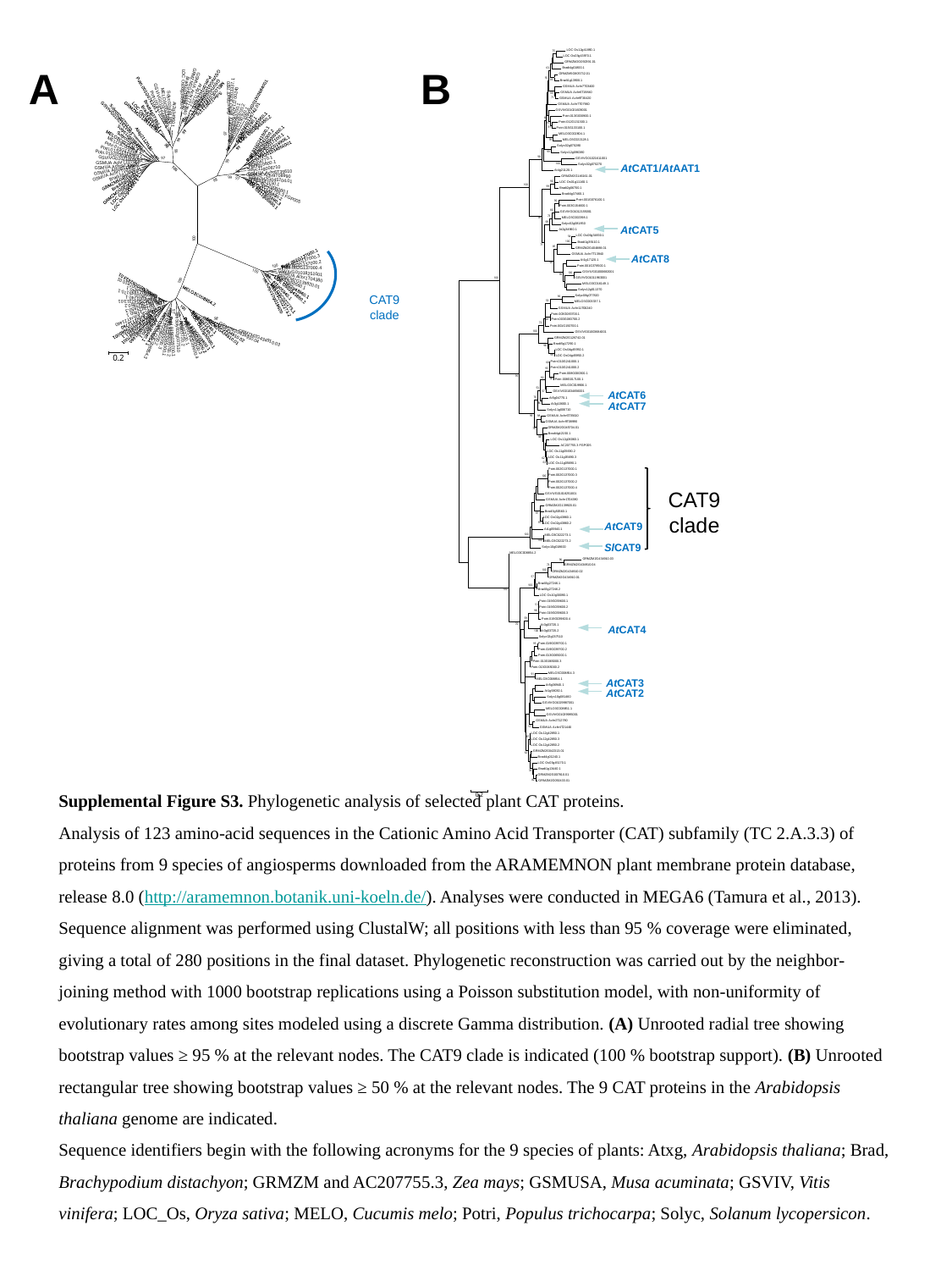

AtCAT9
SlCAT9
CAT9 clade
AtCAT1/AtAAT1
AtCAT5
AtCAT8
AtCAT6
AtCAT7
AtCAT4
AtCAT3
AtCAT2
A
B
CAT9
clade
Supplemental Figure S3. Phylogenetic analysis of selected plant CAT proteins.
Analysis of 123 amino-acid sequences in the Cationic Amino Acid Transporter (CAT) subfamily (TC 2.A.3.3) of proteins from 9 species of angiosperms downloaded from the ARAMEMNON plant membrane protein database, release 8.0 (http://aramemnon.botanik.uni-koeln.de/). Analyses were conducted in MEGA6 (Tamura et al., 2013). Sequence alignment was performed using ClustalW; all positions with less than 95 % coverage were eliminated, giving a total of 280 positions in the final dataset. Phylogenetic reconstruction was carried out by the neighbor-joining method with 1000 bootstrap replications using a Poisson substitution model, with non-uniformity of evolutionary rates among sites modeled using a discrete Gamma distribution. (A) Unrooted radial tree showing bootstrap values ≥ 95 % at the relevant nodes. The CAT9 clade is indicated (100 % bootstrap support). (B) Unrooted rectangular tree showing bootstrap values ≥ 50 % at the relevant nodes. The 9 CAT proteins in the Arabidopsis thaliana genome are indicated.
Sequence identifiers begin with the following acronyms for the 9 species of plants: Atxg, Arabidopsis thaliana; Brad, Brachypodium distachyon; GRMZM and AC207755.3, Zea mays; GSMUSA, Musa acuminata; GSVIV, Vitis vinifera; LOC_Os, Oryza sativa; MELO, Cucumis melo; Potri, Populus trichocarpa; Solyc, Solanum lycopersicon.
