# Supplementary material for: A tonoplast Glu/Asp/GABA exchanger that affects tomato fruit amino acid composition
Source: Plant J. 2015 Feb 24;81(5):651–60. doi: 10.1111/tpj.12766 (PMC4950293; doi:10.1111/tpj.12766)
Supplement: Supplementary file 7 — Figure S4. Quantification and localisation of SlCat9 by western blotting. [file TPJ-81-651-s007.pptx]

## Slide 1
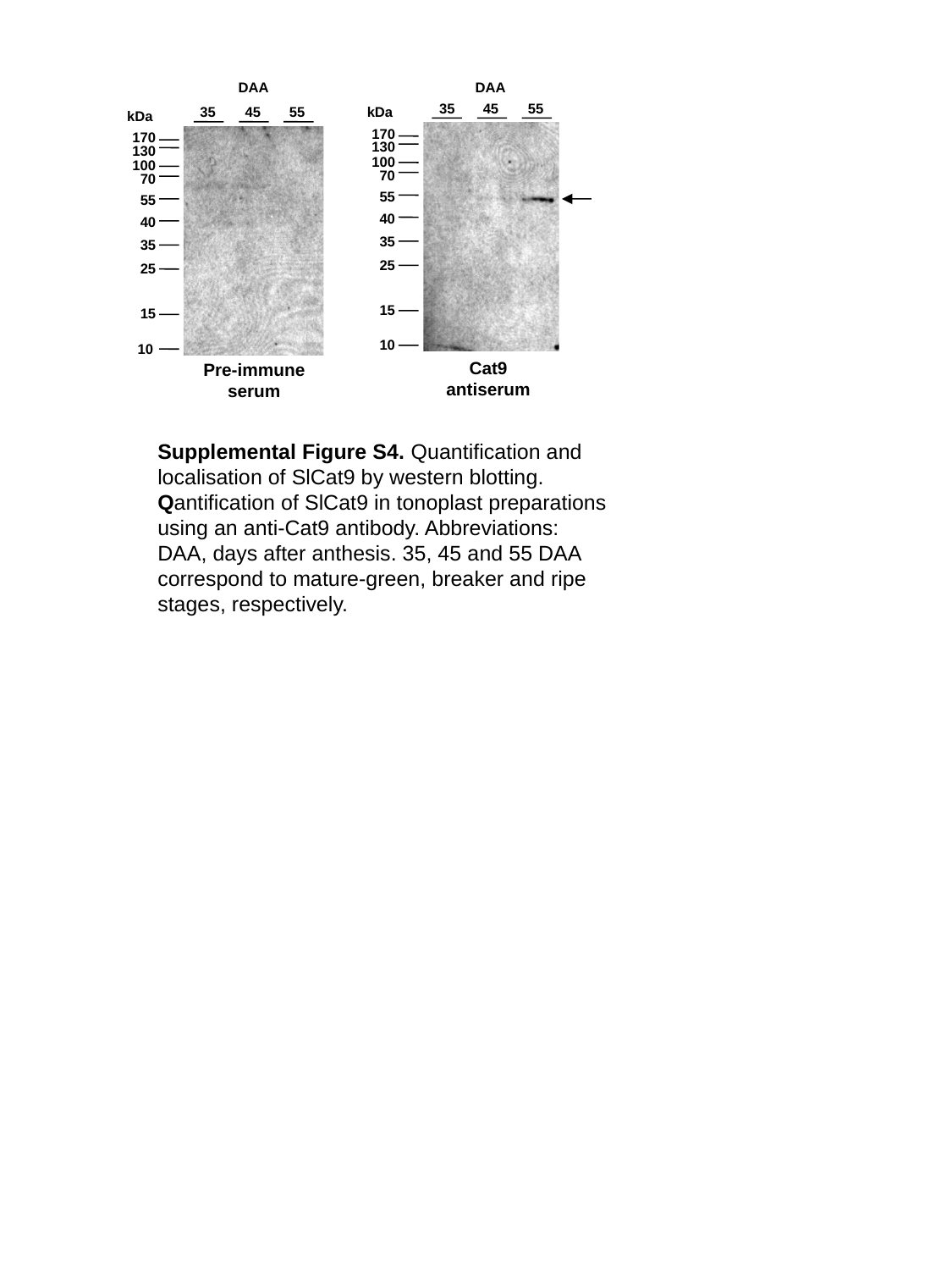

DAA
DAA
35
45
55
35
45
55
kDa
kDa
170
170
130
130
100
100
70
70
55
55
40
40
35
35
25
25
15
15
10
10
Cat9 antiserum
Pre-immune serum
Supplemental Figure S4. Quantification and localisation of SlCat9 by western blotting.
Qantification of SlCat9 in tonoplast preparations using an anti-Cat9 antibody. Abbreviations: DAA, days after anthesis. 35, 45 and 55 DAA correspond to mature-green, breaker and ripe stages, respectively.
