# Supplementary material for: A tonoplast Glu/Asp/GABA exchanger that affects tomato fruit amino acid composition
Source: Plant J. 2015 Feb 24;81(5):651–60. doi: 10.1111/tpj.12766 (PMC4950293; doi:10.1111/tpj.12766)
Supplement: Supplementary file 8 — Figure S5. Characterisation of transgenic tomato plants expressing SlCat9YFP under the control of the ethylene‐inducible E8 promoter. [file TPJ-81-651-s008.pptx]

## Slide 1
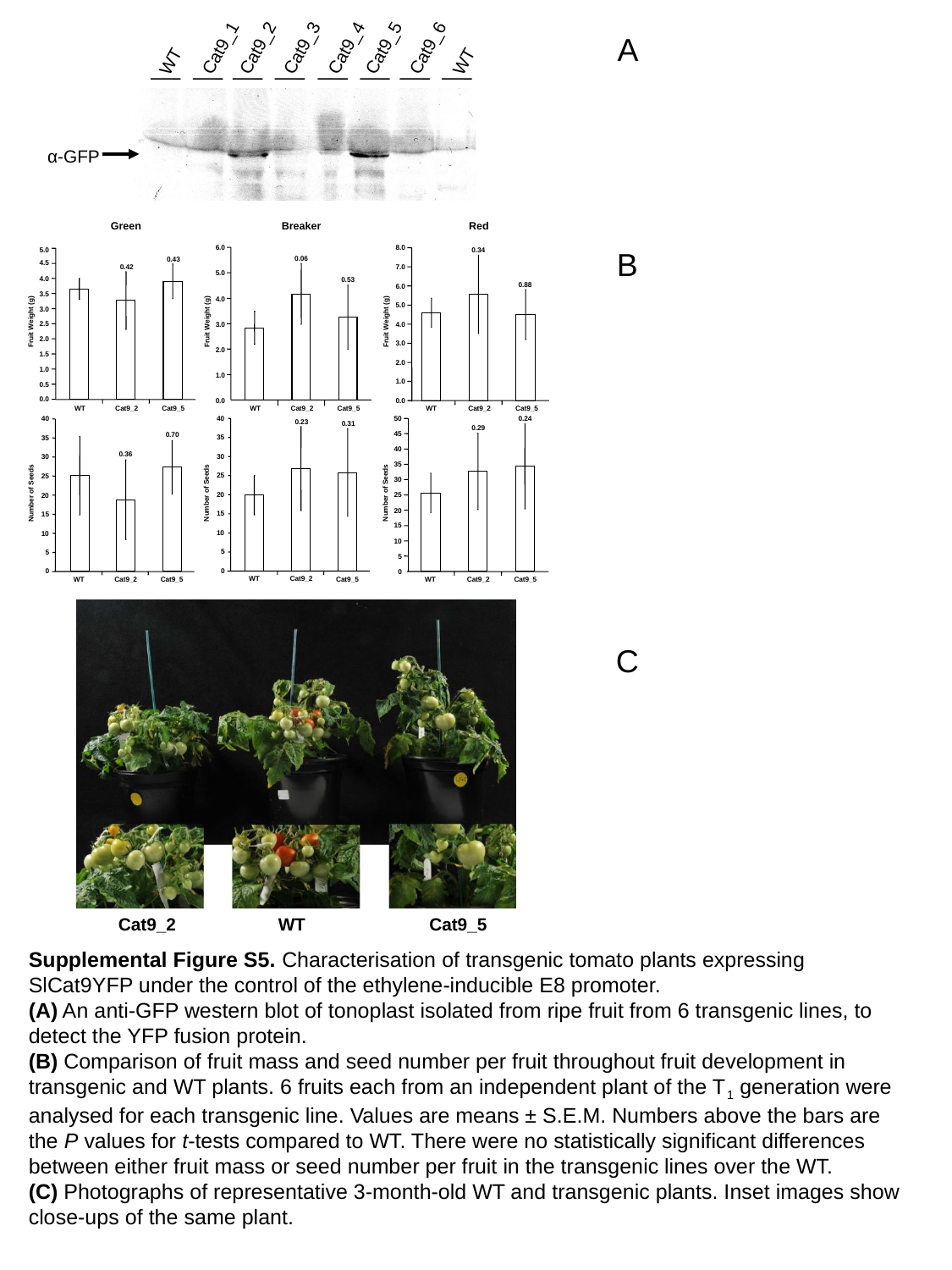

A
Cat9_1
Cat9_2
Cat9_3
Cat9_4
Cat9_5
Cat9_6
WT
WT
α-GFP
Green
Breaker
Red
B
6.0
8.0
5.0
0.34
0.06
0.43
4.5
0.42
7.0
5.0
4.0
0.53
0.88
6.0
3.5
4.0
5.0
3.0
Fruit Weight (g)
Fruit Weight (g)
Fruit Weight (g)
2.5
3.0
4.0
2.0
3.0
2.0
1.5
2.0
1.0
1.0
1.0
0.5
0.0
0.0
0.0
WT
Cat9_2
Cat9_5
WT
Cat9_2
Cat9_5
WT
Cat9_2
Cat9_5
40
50
40
0.24
0.23
0.31
0.29
45
0.70
35
35
40
0.36
30
30
35
25
25
30
Number of Seeds
Number of Seeds
Number of Seeds
20
25
20
20
15
15
15
10
10
10
5
5
5
0
0
0
WT
Cat9_2
Cat9_5
WT
Cat9_2
Cat9_5
WT
Cat9_2
Cat9_5
C
Cat9_2
WT
Cat9_5
Supplemental Figure S5. Characterisation of transgenic tomato plants expressing SlCat9YFP under the control of the ethylene-inducible E8 promoter.
(A) An anti-GFP western blot of tonoplast isolated from ripe fruit from 6 transgenic lines, to detect the YFP fusion protein.
(B) Comparison of fruit mass and seed number per fruit throughout fruit development in transgenic and WT plants. 6 fruits each from an independent plant of the T1 generation were analysed for each transgenic line. Values are means ± S.E.M. Numbers above the bars are the P values for t-tests compared to WT. There were no statistically significant differences between either fruit mass or seed number per fruit in the transgenic lines over the WT.
(C) Photographs of representative 3-month-old WT and transgenic plants. Inset images show close-ups of the same plant.
