# Supplementary material for: A tonoplast Glu/Asp/GABA exchanger that affects tomato fruit amino acid composition
Source: Plant J. 2015 Feb 24;81(5):651–60. doi: 10.1111/tpj.12766 (PMC4950293; doi:10.1111/tpj.12766)
Supplement: Supplementary file 9 — Figure S6. Quantification of SlCAT9‐YFP transgene expression during fruit development by YFP fluorescence. [file TPJ-81-651-s009.pptx]

## Slide 1
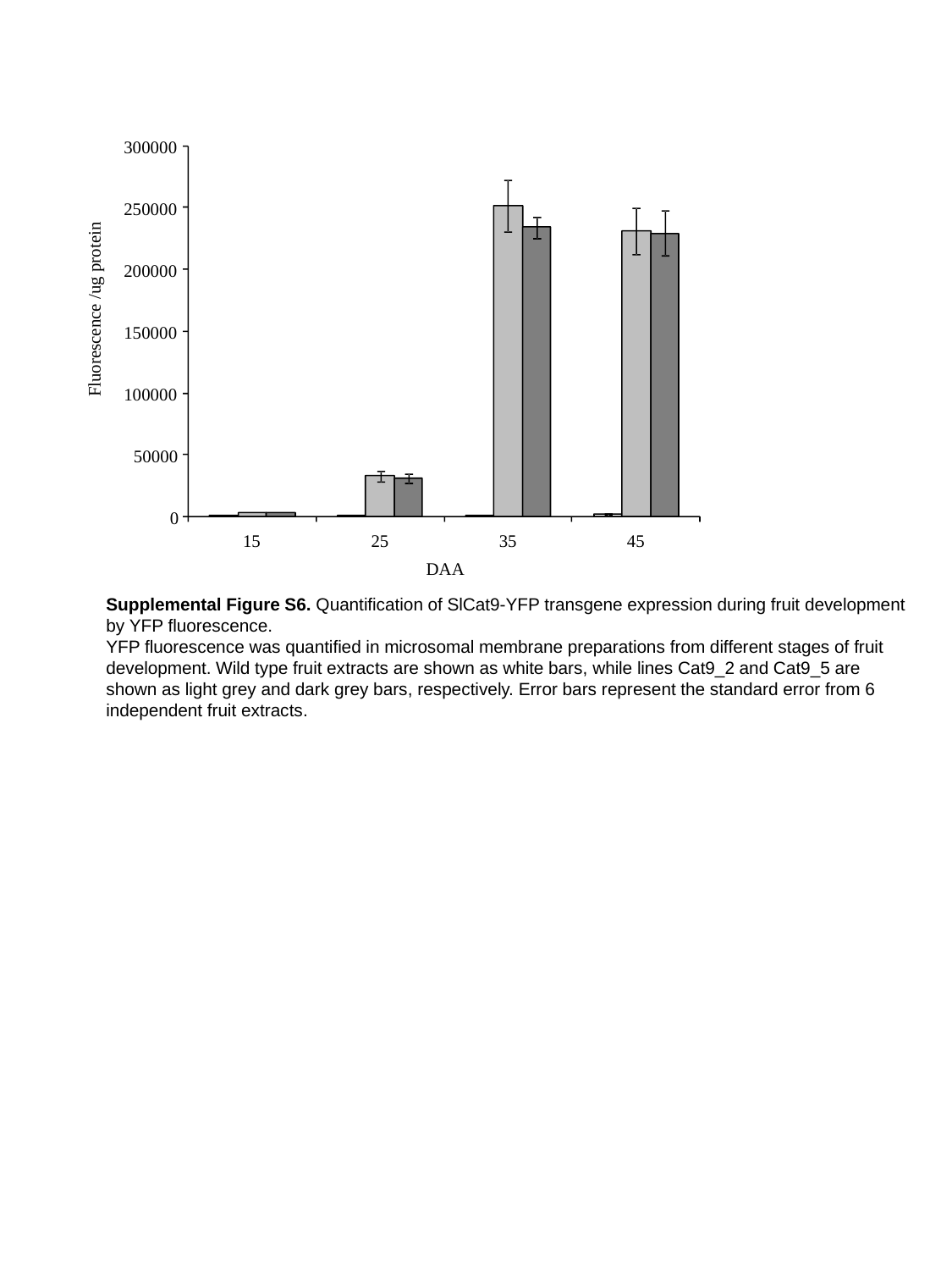

300000
250000
200000
Fluorescence /ug protein
150000
100000
50000
0
15
25
35
45
DAA
Supplemental Figure S6. Quantification of SlCat9-YFP transgene expression during fruit development by YFP fluorescence.
YFP fluorescence was quantified in microsomal membrane preparations from different stages of fruit development. Wild type fruit extracts are shown as white bars, while lines Cat9_2 and Cat9_5 are shown as light grey and dark grey bars, respectively. Error bars represent the standard error from 6 independent fruit extracts.
