# Supplementary material for: A tonoplast Glu/Asp/GABA exchanger that affects tomato fruit amino acid composition
Source: Plant J. 2015 Feb 24;81(5):651–60. doi: 10.1111/tpj.12766 (PMC4950293; doi:10.1111/tpj.12766)
Supplement: Supplementary file 10 [file TPJ-81-651-s010.docx]

**Supporting Information Legends**

**Supplemental Figure S1.** Changes of Key Acidic Metabolites during Fruit Development. Metabolites were quantified in pericarp tissue of *Solanum lycopersicum* (cultivar M82) fruit at the indicated number of days after anthesis. Values are the means of 6 fruit from independent plants ± SE.

**Supplemental Figure S2.** Assessment of purity of tonoplast membrane fractions. Effect of inhibitors of specific classes of ATPase. Nitrate was used to inhibit tonoplast V-ATPase, vanadate to inhibit plasma membrane P-ATPase, and azide to inhibit F-ATPase. The percentage inhibition of ATPase activity (measured as production of P_i_) is shown for fractions taken from the indicated percentage sucrose steps of a stepped sucrose density gradient and the pelleted material that passed through the gradient. Membranes from pericarp of fruit from three developmental stages (at the indicated number of days after anthesis, DAA) were fractionated. The fraction collected from the 6-12% sucrose interface proved to be a good balance between yield and purity. (B) SDS-PAGE of membrane proteins collected from the 6–12% (w/v) sucrose interface and carbonate washed. 15 μg of protein were loaded per lane. (C) Effect of the specific inhibitor of the tonoplast ATPase, Concanamycin A (Con A), on ATP-dependent proton pumping into tonoplast vesicles from the 6-12% sucrose interface. ATP-dependent proton pumping was assayed using the quinacrine fluorescence-quenching technique White and Smith (1989) in a medium containing 50 mM BTP-fumarate (pH 8.0) in the presence or absence of 1 μM concanamycin A as indicated. The activity of the control in the absence of inhibitor (100%) corresponded to a relative fluorescence quench of 389% min^−1^ mg protein^−1^. White PJ & Smith JAC (1989) Proton and anion transport at the tonoplast in crassulacean-acid-metabolism plants: specificity of the malate-influx system in *Kalanchoë daigremontiana*. Planta 179:265–274.

**Supplemental Figure S3.** Phylogenetic analysis of selected plant CAT proteins. Analysis of 123 amino-acid sequences in the Cationic Amino Acid Transporter (CAT) subfamily (TC 2.A.3.3) of proteins from 9 species of angiosperms downloaded from the ARAMEMNON plant membrane protein database, release 8.0 (<http://aramemnon.botanik.uni-koeln.de/>). Analyses were conducted in MEGA6 (Tamura et al., 2013). Sequence alignment was performed using ClustalW; all positions with less than 95 % coverage were eliminated, giving a total of 280 positions in the final dataset. Phylogenetic reconstruction was carried out by the neighbor-joining method with 1000 bootstrap replications using a Poisson substitution model, with non-uniformity of evolutionary rates among sites modeled using a discrete Gamma distribution. **(A)** Unrooted radial tree showing bootstrap values ≥ 95 % at the relevant nodes. The CAT9 clade is indicated (100 % bootstrap support). **(B)** Unrooted rectangular tree showing bootstrap values ≥ 50 % at the relevant nodes. The 9 CAT proteins in the *Arabidopsis thaliana* genome are indicated. Sequence identifiers begin with the following acronyms for the 9 species of plants: Atxg, *Arabidopsis thaliana*; Brad, *Brachypodium distachyon*; GRMZM and AC207755.3, *Zea mays*; GSMUSA, *Musa acuminata*; GSVIV, *Vitis vinifera*; LOC_Os, *Oryza sativa*; MELO, *Cucumis melo*; Potri, *Populus trichocarpa*; Solyc, *Solanum lycopersicon*.

**Supplemental Figure S4.** Quantification and localisation of SlCat9 by western blotting. **Q**antification of SlCat9 in tonoplast preparations using an anti-Cat9 antibody. Abbreviations: DAA, days after anthesis. 35, 45 and 55 DAA correspond to mature-green, breaker and ripe stages, respectively.

**Supplemental Figure S5.** Characterisation of transgenic tomato plants expressing SlCat9YFP under the control of the ethylene-inducible E8 promoter. **(A)** An anti-GFP western blot of tonoplast isolated from ripe fruit from 6 transgenic lines, to detect the YFP fusion protein. **(B)** Comparison of fruit mass and seed number per fruit throughout fruit development in transgenic and WT plants. 6 fruits each from an independent plant of the T_1_ generation were analysed for each transgenic line. Values are means ± S.E.M. Numbers above the bars are the *P* values for *t*-tests compared to WT. There were no statistically significant differences between either fruit mass or seed number per fruit in the transgenic lines over the WT. **(C)** Photographs of representative 3-month-old WT and transgenic plants. Inset images show close-ups of the same plant.

**Supplemental Figure S6.** Quantification of SlCat9-YFP transgene expression during fruit development by YFP fluorescence. YFP fluorescence was quantified in microsomal membrane preparations from different stages of fruit development. Wild type fruit extracts are shown as white bars, while lines Cat9_2 and Cat9_5 are shown as light grey and dark grey bars, respectively. Error bars represent the standard error from 6 independent fruit extracts.

**Supplemental Table S1.** Integral membrane tonoplast proteins identified by proteomic analysis of isolated tonoplast-enriched membrane fractions from tomato fruit.

**Supplemental Table S2.** Metabolite content of ripe transgenic tomato fruit overexpressing SlCAT9. Ripe fruit (harvested at 38 days after anthesis) from wild type and T2-generation transgenic Micro=Tom plants were harvested and pericarp samples taken. For each sample, 4 fruits were pooled from a single plant. Values shown are the means ± SE of 6 samples, each taken from an independent plant. WT, wild type; nd, not detectable. Values in bold are significantly different from WT (t-test; P < 0.05).

**Supplemental Data S1.** Shotgun proteomics of tonoplast-enriched membrane fractions from tomato fruit.
